# Camouflaging endovascular stents with an endothelial coat using CD31 domain 1 and 2 mimetic peptides

**DOI:** 10.1016/j.jvssci.2024.100213

**Published:** 2024-07-23

**Authors:** Jean Sénémaud, Charles Skarbek, Belen Hernandez, Ran Song, Isabelle Lefevre, Elisabetta Bianchi, Yves Castier, Antonino Nicoletti, Christophe Bureau, Giuseppina Caligiuri

**Affiliations:** a Department of Vascular Surgery, Bichat University Hospital, Paris, France; b Université Paris Cité, Paris, France; c Laboratory for Vascular Translational Science, INSERM U1148, Paris, France; d Université Sorbonne Paris Nord, Paris, France; e Sino Medical Sciences Technology Inc., Tianjin, China; f AlchiMedics S.A.S., Paris, France; g Peptide Chemistry Unit, Peptides & Small Molecules R&D Department, IRBM SpA, Pomezia, Roma, Italy; h Department of Cardiology, Bichat University Hospital, Paris, France

**Keywords:** CD31 interactions, Endovascular stents, Camouflage strategy, Circulatory homeostasis, Device integration

## Abstract

**Objective:**

Implantation of an endovascular device disrupts the homeostatic CD31:CD31 interactions among quiescent endothelial cells (ECs), platelets, and circulating leukocytes. The aim of this study was to design an endothelial-mimetic coating of nitinol and cobalt-chromium (CoCr) surfaces and stents using synthetic CD31 peptides, to promote device endothelialization and pacific integration within the arterial wall.

**Methods:**

Peptides mimicking the domains 1 (D1) and 2 (D2) of CD31 were synthetized and immobilized onto experimental nitinol and CoCr surfaces using a three-step, dip-coating, mussel-inspired protocol using copper-free click chemistry. Human aortic EC phenotype and endothelialization assessment using parallel scratch tests were carried out using five synthetic CD31 peptides coated on 4.8-mm nitinol and CoCr flat disks and were compared with control disks. The CD31 peptide exhibiting the best results in vitro was then immobilized on clinical-grade 3 × 40-mm self-expanding nitinol and 2.5 × 20.0-mm balloon-expandable CoCr stents. Such devices were implanted in native arteries of White New Zealand rabbits, and compared with control uncoated bare metal stents (BMS) and drug-eluting stents 7 and 30 days after implantation using resin cross-sections and scanning electron microscopy (n = 2-3 per group at each time point).

**Results:**

Membrane-distal CD31 D1 and D2 peptides exhibited a distinct capability to foster a healthy endothelial phenotype and to promote endothelialization in vitro. By day 7 after implantation, CD31 nitinol and CoCr stents were evenly covered by wholesome ECs, devoid of thromboinflammatory signs, in contrast with both BMS and drug-eluting stents. Such results were consistent until day 30.

**Conclusions:**

Membrane-distal CD31 biomimetic peptides seem to camouflage the device surface effectively, preventing local reactions and promoting rapid and seamless endovascular integration.

## Clinical Relevance

Despite significant technical advancements, the challenge of vascular stent healing persists. In this preclinical study, we developed a novel endothelial-mimetic coating inspired by the trans-homophilic portion of CD31, a glycoprotein predominantly expressed by endothelial cells and pivotal in circulatory homeostasis. After successive peptide design and surface functionalization, both in vitro and in vivo experiments demonstrated the potential of the CD31-coated surface to promote the acquisition of a physiological endothelial cell phenotype, facilitating rapid healing and peaceful integration of stents. Further studies with larger sample sizes, extended follow-up periods, and assessment of the impact on in-stent restenosis are essential before translating this strategy to clinical applications.

**Article Highlights**

• **Type of Research:** We conducted an silico, in vitro, and in vivo study of CD31-mimetic peptides
• **Key Findings:** CD31-coated surfaces promoted the acquisition of a physiological endothelial cell phenotype and rapid coverage at a significantly higher extent as compared with bare metallic surfaces in vitro. Nitinol and cobalt-chromium stents coated with CD31-mimetic peptides were integrated rapidly within the arterial wall in vivo without thromboinflammatory signs up to 30 days after implantation.
• **Take Home Message:** A biomimetic CD31 coating might work as an endothelial camouflage for stents, promoting their Pacific integration in the vasculature.

The introduction of endovascular devices during percutaneous arterial interventions disrupts the homeostatic molecular cross-talk between the bloodstream platelets, leukocytes, and vascular endothelial cells (ECs), triggering a pathogenic thromboinflammatory response. The latter includes immediate endothelial damage, early platelet aggregation, and leucocyte infiltration, leading to vascular smooth cells colonization and ultimately to neointima formation,^1^^,^^2^ which strongly impairs long-term results of arterial stenting through in-stent restenosis formation.^3^

Drug-eluting stents (DESs) were developed to mitigate such inflammatory and proliferative responses with satisfactory clinical results compared with bare-metal stents (BMSs) in terms of in-stent restenosis rates.^4^ However, the nonselective cytotoxic effect of released drugs on ECs induces a delayed healing of the stented artery, perpetrating exposure of the device metallic surface to blood clotting factors. Such exposure requires a prolonged regimen of dual antiplatelet therapy (DAPT) postoperatively to counteract the heightened risk of in-stent thrombosis.^5^

ECs are essential for maintaining critical physiological functions within the circulation, including anti-inflammatory and antithrombotic processes. To carry out these functions effectively, ECs depend on appropriate signaling cues. One key element in this process is the abundant expression of CD31, also known as platelet EC adhesion molecule-), by healthy ECs. CD31 is a large type I transmembrane glycoprotein consisting of 738 amino acids with a molecular weight of 130 kDa. It comprises six extracellular immunoglobulin-like domains, a short transmembrane fragment, and a cytoplasmic tail with regulatory signaling properties.

CD31 is located primarily between ECs, where it plays crucial roles in endothelial physiology.^6^ Importantly, CD31 is also expressed by circulating leukocytes and platelets, enabling it to regulate platelet adhesion and activation at the vascular endothelium interface. All such regulatory functions occur through trans-homophilic interactions between domains 1 (D1) and 2 (D2) of CD31 molecules on interacting CD31^+^ cells. The exposure of D1 and D2 of CD31 on the luminal aspect of the endothelium is hence vital for preventing the adhesion and activation of circulating platelets and leukocytes.^6^ The anti-inflammatory and antithrombotic properties of CD31 rely on its co-clusterization with stimulated receptors on the surface of platelets and leukocytes.

The trans-homophilic interaction of CD31 D1 and D2 is crucial not only for providing an active detachment signal to interacting CD31^+^ cells,^7^ but also for maintaining the clusterization of the juxtamembrane extracellular sequence via strong cis-homophilic interaction occurring at sites of cell activation, which is essential for allowing the phosphorylation of the two immunoreceptor tyrosine-based inhibitory motifs present in the cytoplasmic tail of CD31 and enable the regulatory function of CD31 through the recruitment of tyrosine and inositol phosphatases (reviewed by Caligiuri^8^^,^^9^). However, despite CD31 expression being constitutive, it is susceptible to proteolytic shedding triggered by cell membrane proteases upon cell activation. This shedding process results in the loss of the membrane-distal trans-homophilic portion, including D1 and D2, leaving a truncated CD31 primarily consisting of the juxtamembrane extracellular fragment on activated cells.^10^

Previously, we explored the potential of P8RI, a synthetic peptide able to co-oligomerize with the juxtamembrane fragment, thereby stabilizing its clustering in cells undergoing shedding. By mitigating leukocyte and platelet activation upon contact with the device surface, cobalt-chromium (CoCr) and nitinol stents coated with P8RI were less prone to foster the thrombotic and inflammatory response and were more rapidly integrated within the arterial wall.^11^^,^^12^ However, in vitro experiments revealed that the decrease in pro-inflammatory or prothrombotic mediators by healthy ECs when in contact with the experimental surfaces was often similar between P8RI-coated surfaces and surfaces coated only with the nonspecific adhesive used to attach the peptide onto the devices, such as polydopamine (PDA) or polyethylene glycol.^11^ The relatively minimal specific effect of P8RI observed in vitro likely stems from the concealed nature of the target sequence within the intact, large extracellular CD31 structure, which is exposed by healthy cells and likely prevents the interaction between surface-bound P8RI and the homotypic juxtamembrane sequence of whole CD31 molecules.

In this work, our objective was to explore another strategy: harnessing the characteristics of CD31 membrane-distal D1 and D2 to camouflage endovascular devices. We hypothesized that this strategy could enable stents to be perceived as integral components of the healthy endothelium, potentially facilitating their recognition as part of the physiological environment by cells interacting with their surfaces. Consequently, this approach could provide a trans-homophilic CD31 platform to adjacent CD31^+^ cells, thereby promoting seamless integration within the cardiovascular system.

## Methods

### Biomimicking peptides

The biomimicking peptides inspired by human CD31 trans-homophilic interactions were designed based on the structural characterization of D1 and D2 and their strand-swapped dimer formation observed on CD31 crystallization, as described by Paddock et al.^13^ The substantial D1/D1 interface and interdomain contacts between D1/D2 and D2/D2 provided critical information to inform the development of these peptides, with the aim of enhancing and replicating the cell-cell trans-homophilic interactions crucial for CD31 intercellular functions. The designed peptides were divided into four groups, referred to as group I, group II, group III, and group IVa/b. Peptide design, synthesis, and structural analysis are described in the Supplementary Methods.

### Surface functionalization and peptide coating

The coating process for all samples, including nitinol and CoCr disks and stents, was achieved following a three-step protocol previously described.^11^^,^^12^ We ensured coating quality control by conducting X-ray photoelectron spectroscopy analysis and high-magnification three-dimensional (3D) light microscopy inspection (VHX-7000N, Osaka, Japan). This was done before and after resheating and re-expansion of parallel samples from each batch to confirm coating stability during procedural maneuvers. Additionally, high-magnification 3D light microscopy inspection was performed on every individual experimental sample.

### ECs phenotype and endothelialization assessment

Distinct CD31 peptide coatings impact on primary human aortic ECs (HAECs; Lonza, Basel, Switzerland) was assessed through in vitro screening. Biomimicking peptides underwent batch testing, and individual peptide effects were assessed across multiple experiments. Because PDA may itself have a biological response, we used both bare-metal and PDA-coated samples as control groups for all experiments along with a peptide targeting the cis-homophilic, juxta-membrane segment, of CD31 (P8RI).^11^^,^^12^

Mirror-polished nitinol disks (4.8-mm diameter, 0.15-mm thickness; Goodfellow Cambridge Ltd, Huntingdon, UK) underwent sequential dip coating as described. Primary HAECs were cultured on these disks for 48 hours (25,000 cells/disk) using basal Endothelial Cell Basal Medium MV2 (Lonza), excluding growth factors.

After incubation, adherent cells on disks were rinsed, fixed, and stained. Staining included phalloidin-AlexaFluor 488 for F-actin, DAPI for DNA, and CD31 D1 antibody (clone 9G11)-allophycocyanin for CD31. Fluorescent elements were imaged using an Axio Observer microscope with Zeiss Zen software (Zeiss, Jena, Germany) and analyzed using Image J (National Institutes of Health, Bethesda, MD) with the "Analyze Particles" function.

Adherent elements were quantified as N/mm^2^, and positive staining was assessed via integrated density. A physiological endothelial phenotype was indicated by low intracellular F-actin and strong CD31 expression at cell borders, presented as CD31/Actin ratios on a Y-axis scaled from 0 to 1 using the formula (x − min)/(max − min).

To assess the impact of different CD31 peptide coatings on endothelialization potential comprehensively, parallel scratch tests were performed. Coatings were applied to culture well bottoms using the previously described method using the SP1374 (group I), SP1072 (group II), SP1380 (group III), and P8RI peptides onto 96-well plates for use in the scratch test using the previously described three steps, and real-time cell migration was tracked using an Incucyte S5 system (Sartorius, Gottingen, Germany), providing insights into the influence of various CD31 peptide coatings on EC migration.

### Stent implantation and evaluation

For this initial investigation, we prioritized qualitative evaluation over statistical analysis aiming for consistent longitudinal assessment across two distinct device types: nitinol self-expanding and CoCr balloon-expandable devices. Our approach aimed to maximize information extraction while adhering to the principles of the 3 *R*s (replacement, reduction, and refinement) to minimize animal use.

The peptide SP1072 (group II, sequence: YKSTVIVNNKEKTTAE-PEG4-K(N3)-CONH2), chosen from those exhibiting a robust CD31/actin ratio in vitro along with HAEC confluence and growth potential, was used to coat clinical-grade 3 × 40-mm self-expanding nitinol flow diverters and 2.5 × 20-mm balloon-expandable CoCr stents (all stents were provided by Sino Medical Sciences Technology Inc., Tianjin, China).

The in vivo studies were carried out in male White New Zealand rabbits weighing 4.0 to 4.5 kg and comprised two arms. In the first arm, six rabbits were implanted with two nitinol flow diverters (either bare or SP1072 coated), one in the innominate artery and one in the abdominal aorta. Flow diverter devices consisted of interwoven 35 NLT DFT alloy wires and were delivered using a dedicated 2.4 F Headaway 21 microcatheter (MicroVention Inc., Terumo, CA). Evaluation was conducted at different time points: days 7, 30, and 60.

In the second arm, six rabbits received four different types of balloon-expandable CoCr stents. Each rabbit was implanted with one of each stent type: DES (sirolimus 1.2 μg/mm^2^), SP1072-coated stents, BMS, and stents coated with PDA. Evaluation occurred at days 7, 14, and 30.

Stents were implanted to maintain a 1:1 ratio with the dimensions of the target arteries (aorta and iliac arteries), ensuring that stent expansion was sufficient for stable implantation while avoiding overexpansion of the arterial segments. The stent implantation procedures and postoperative care are detailed in the Supplementary Methods. All in vivo procedures described in this study were conducted in compliance with the Principles of Laboratory Animal Care 14 and were approved by the local Institutional Animal Care and Use Committee (#PLJC22-0005, PharmaLegacy Diagnostics Co. Ltd, Shanghai, People's Republic of China).

Histopathology analysis on resin cross-sections and en face scanning electron microscopy analyses were conducted at each time points, following established protocols^11^^,^^12^ and are detailed in the Supplementary Methods.

### Statistical analysis

Data are presented as mean ± standard deviation. The Kruskal-Wallis test with Dunn post-test was used for skewed data. Normally distributed variables were analyzed with one-way analysis of variance with Bonferroni post hoc F. The Fisher exact test with Bonferroni correction for multiple tests was used for comparison of nominal variables. All statistical calculations were performed with R software (R-3.6.1; The R Foundation for Statistical Computing, Vienna, Austria). Differences were considered statistically significant at a *P* value of <.05.

## Results

### Synthetic peptides and surface characterization

**Supplementary Fig 1 (online only):**
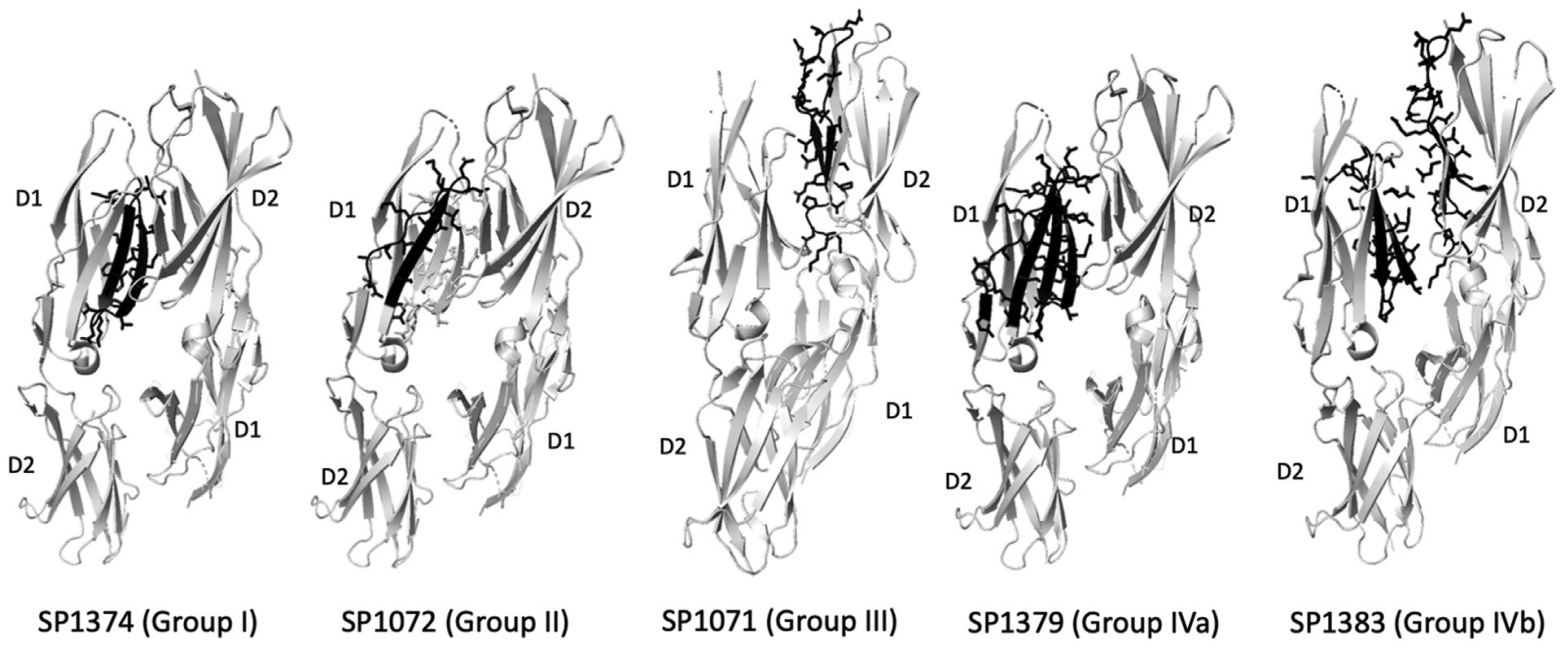
Localization of representative peptides within CD31 domains 1 (D1) and 2 (D2). The positioning of representative peptides used in this study is illustrated within the trans-homophilic interacting sequences of CD31 D1 and D2. The CD31 protein sequence is presented in grey, while the peptide sequence is depicted in black. Groups I and II comprise peptides issued from D1. Group III comprises peptides issued from D2. Group IV includes dimers comprised either within D1 (group IVa) or situated between D1 of one CD31 molecule and the trans-homophilic segment of D2 of another CD31 molecule (group IVb).

**Supplementary Fig 2 (online only):**
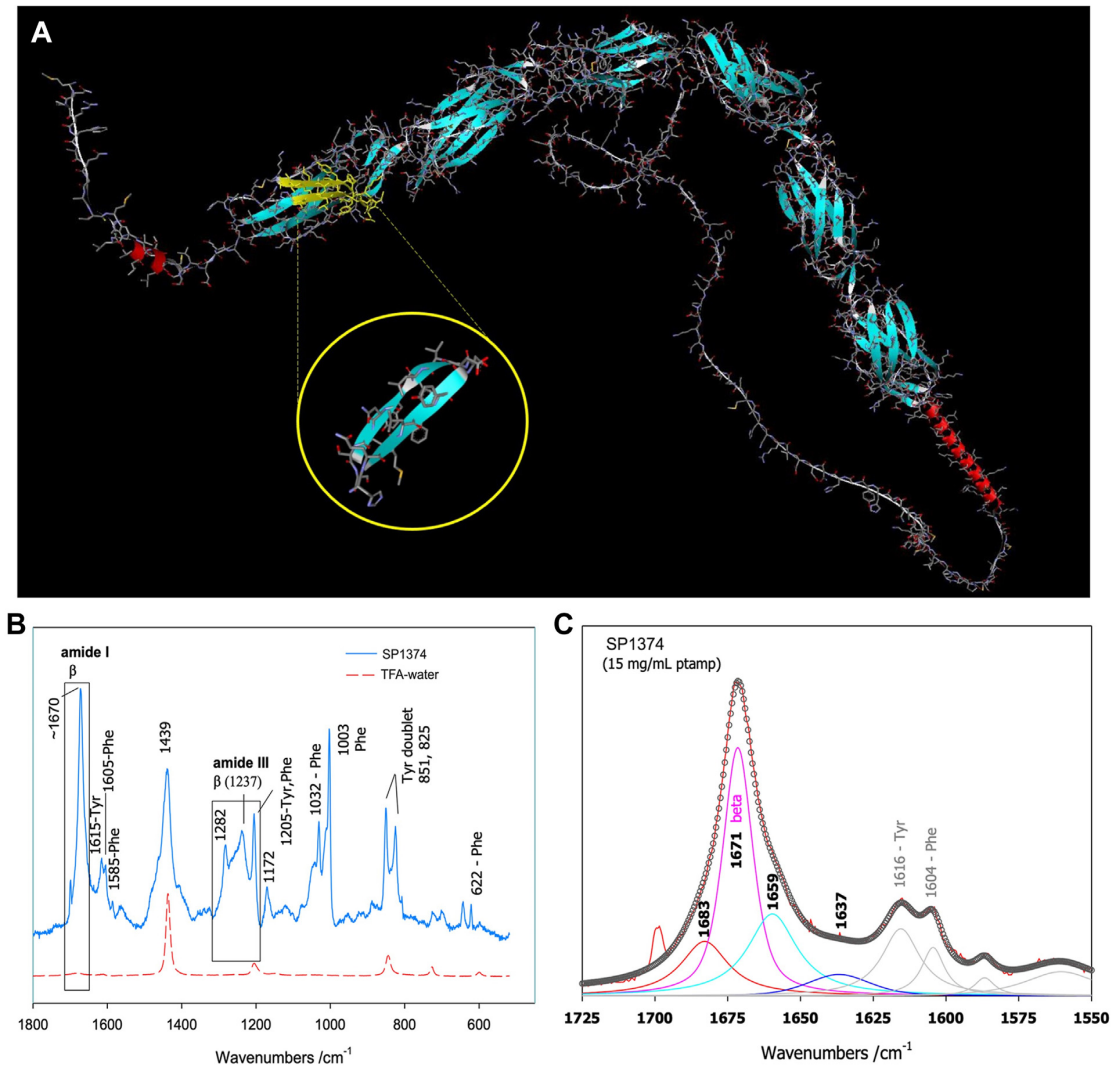
Structural analysis of synthetic peptides via Raman spectroscopy. Representative example (SP1374) of peptide structural data obtained through Raman Spectroscopy. (**A**) Depicts the conformation of the peptide (highlighted within the yellow circle) as compared with its structure within the three-dimensional model of human PECAM-1 (available at https://alphafold.com/entry/P16284). (**B**) Raman spectra in peptide solutions after solvent subtraction, with the trifluoroacetic acid-water spectrum plotted in red. The regions used for structural diagnostics (amides I and III) are outlined (boxes), along with characteristic vibrations of aromatic amino acid side chains. (**C**) Decomposition of the amide I region of peptide SP1374, revealing a highly structured species primarily composed of beta-type conformers organized in sheets (approximately 45%). The remaining contributions correspond with elements that facilitate connections between beta strands (beta-turns), segments adopting PPII conformations, 3 to 10 helices, or disordered segments at the chain termini.

Fifty-one peptides across the five groups were synthetized and tested in preliminary experiments (data not shown) and the best hits (SP1374 from group I, SP1072 from group II, SP1071 from group III, SP1379 from group IVa, SP1383 from group IVb) were used for the present study. The sequence of these peptides were modeled in silico based on the CD31 D1 and D2 trans-dimerization interface model described by Hu et al.^14^ and details are provided in Table I. Their respective position at the trans-homophilic interface of CD31-CD31 dimers is illustrated in the Supplementary Fig 1 (online only) and an example of their structural characterization using Raman spectroscopy is presented in the Supplementary Fig 2 (online only).

**Table I:** Details of the synthetic peptides

Peptide (group)	Protein region	CD31 domain	Sequence
SP1374 (I)	Gln70-Lys89	1	QHQMLFYKDDVLFYNISSMK-Ttds-Ttds-Ttds-Ttds-KN3-CONH2
SP1072 (II)	Tyr107-Glu122	1	YKSTVIVNNKEKTTAE-PEG4-K(N3)-NH2
SP1071 (III)	Pro133-Lys158	2	Ac-K(N3)-PEG4-PRVTLDKKEAIQGGIVRVNSSVPEEK-CONH2
SP1379 (IVa)	Thr106-Gln124 + Gln70-Lys89 (K8hC)	1 (dimer)^a^	[-CH2CO^a^-GTYKSTVIVNNKEKTTAEYQ-(Ttds)4-K(N3)-NH2]-[QHQMLFY-hC^a^-DDVLFYNISSMK-NH2]
SP1383 (IVb)	Pro133-Lys158 + K8hCGln70-Lys89	1-2 (dimer)^a^	[Ac-K(N3)-(Ttds)4-PRVTLDKKEAIQGGIVRVNSSVPEEK-Ttds-K(-CH2CO^a^)NH2]-[QHQMLFY-hC^a^-DDVLFYNISSMK-NH2]

a Dimers are obtained through a thioether linkage of the first sequence with the bromoacetyl group of the second sequence.

The chemical characterization of functionalized surfaces at different steps of the coating process is detailed in Table II, showing successive modification of surface stoichiometry. After deposition of the PDA self-assembled layer, coated surfaces showed strong carbon (C) and nitrogen (N) peaks, in accordance with independent previous studies.^15^ Further addition of the coating over the PDA layer resulted in an increase of the O/C and the N/C ratios, reflecting the CH₂-CH₂O-repeating units of the polyethylene glycol spacer and peptide fixation, respectively.

**Table II:** X-ray photoelectron spectroscopy analysis of surface composition of bare and SP1072-coated nitinol stents

	Bare surface	PDA	SP1072 peptide coating
C	27.3	80.4 ± 0.7	73. 4 ± 1.4
O	58.8	14.1 ± 0.6	19.5 ± 1.1
N	2.1	5.0 ± 0.6	7.4 ± 0.8
S	NA	NA	0.2 ± 0.06
Cr	3.6	0.4 ± 0.02	0.5 ± 0.02
Ni	8.1	NA	NA
O/C ratio	2.15	0.17	0.26
N/C ratio	0.08	0.06	0.10

*C,* Carbon; *Cr,* chromium; *DBCO,* dibenzocyclooctyne; *N,* nitrogen; *NA,* not applicable; *Ni,* nitinol; *O,* oxygen; *PDA,* polydopamine; *S,* sulfur; *XPS,* X-ray photoelectron spectroscopy.

Compositions are presented in percentages (mean ± standard deviation).

### Interaction of ECs with experimental nitinol disks and endothelialization assessment

**Fig 1:**
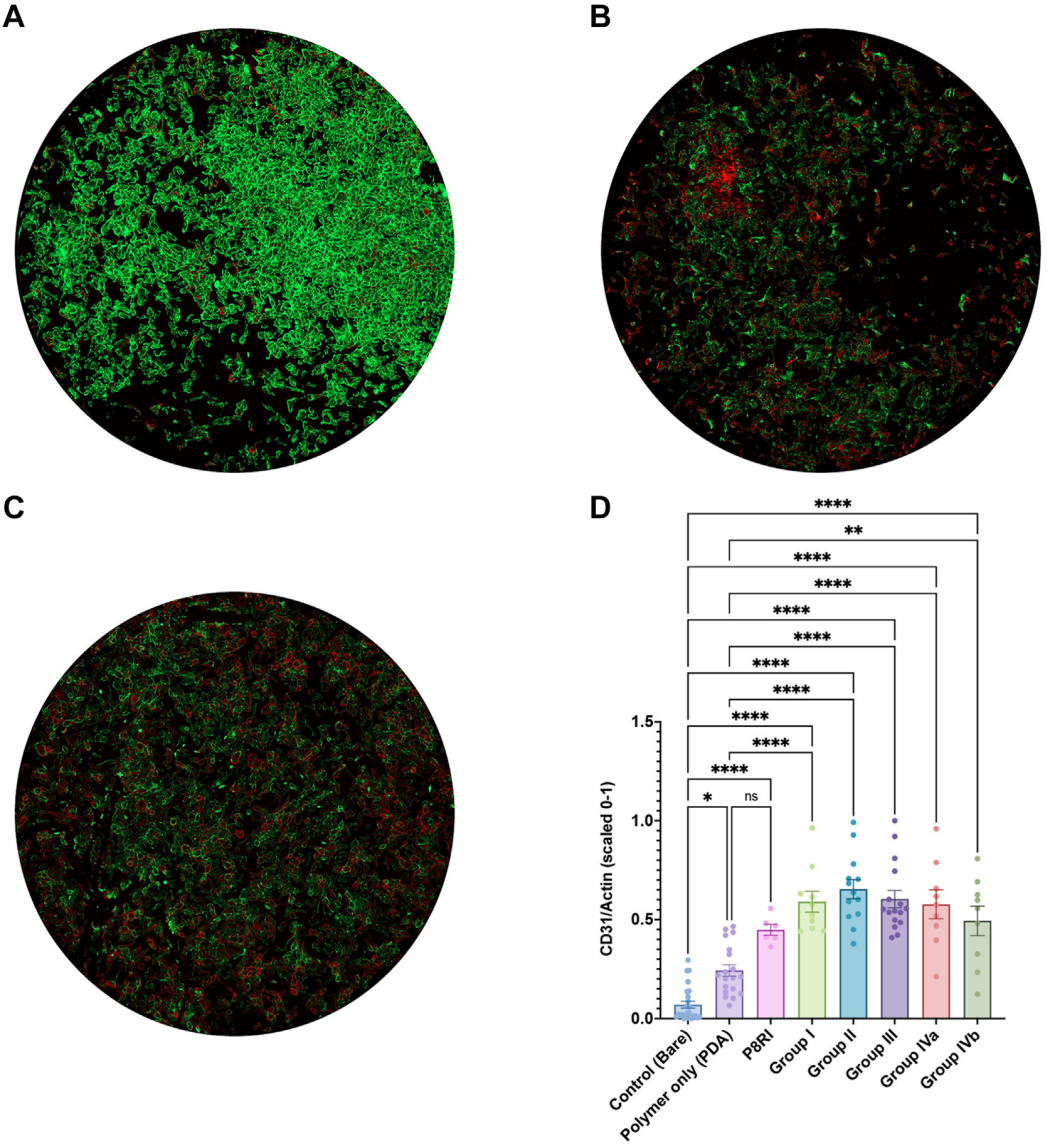
Assessment of endothelial cell (EC) phenotype: CD31/actin ratio. **(A)** Human aortic ECs (HAECs) adhering to bare metal surfaces exhibit a stressed phenotype characterized by extensive F-actin polymerization (green staining) and relatively low CD31 expression at the cell border. **(B)** Introduction of the antifouling and proadhesive polymer, polydopamine (*PDA*), improves the physiological score (CD31/actin ratio) compared with bare metal support. However, CD31 expression remains uneven and variable in density across the endothelial layer. **(C)** The presence of trans-homophilic peptides derived from domain 1 (D1) of CD31 (illustrated here by SP1072 from group II) consistently induces a physiological and uniform phenotype in contacting ECs throughout the flat sample. **(D)** Statistical analysis shows differences between various peptides (pooled data from 2 to 3 batches, including data from 3 to 5 peptides per group, and P8RI) compared with bare metal and discs coated with the polymer only (PDA). *ns*, not significant. ∗*P* < .05, ∗∗*P* < .01, ∗∗∗*P* < .001.

All CD31 peptides consistently promoted a more physiological EC phenotype compared with bare disks in the in vitro testing. Specifically, the CD31/actin ratio of the trans-homophilic peptides from D1 and D2 were significantly increased compared with bare metal disks and PDA-coated disks (Fig 1), whereas the control, cis-homophilic peptide from the juxtamembrane segment (P8RI) of CD31, did not provide a significant difference compared with the polymer alone (PDA; *P* = .21). This finding suggests that, in this assay using resting ECs, the target interactive position of P8RI with the juxtamembrane portion of the whole CD31 expressed by the healthy cells was likely obscured by the large mass of the six extracellular immunoglobulin-like domains of CD31 (steric hindrance).

**Fig 2:**
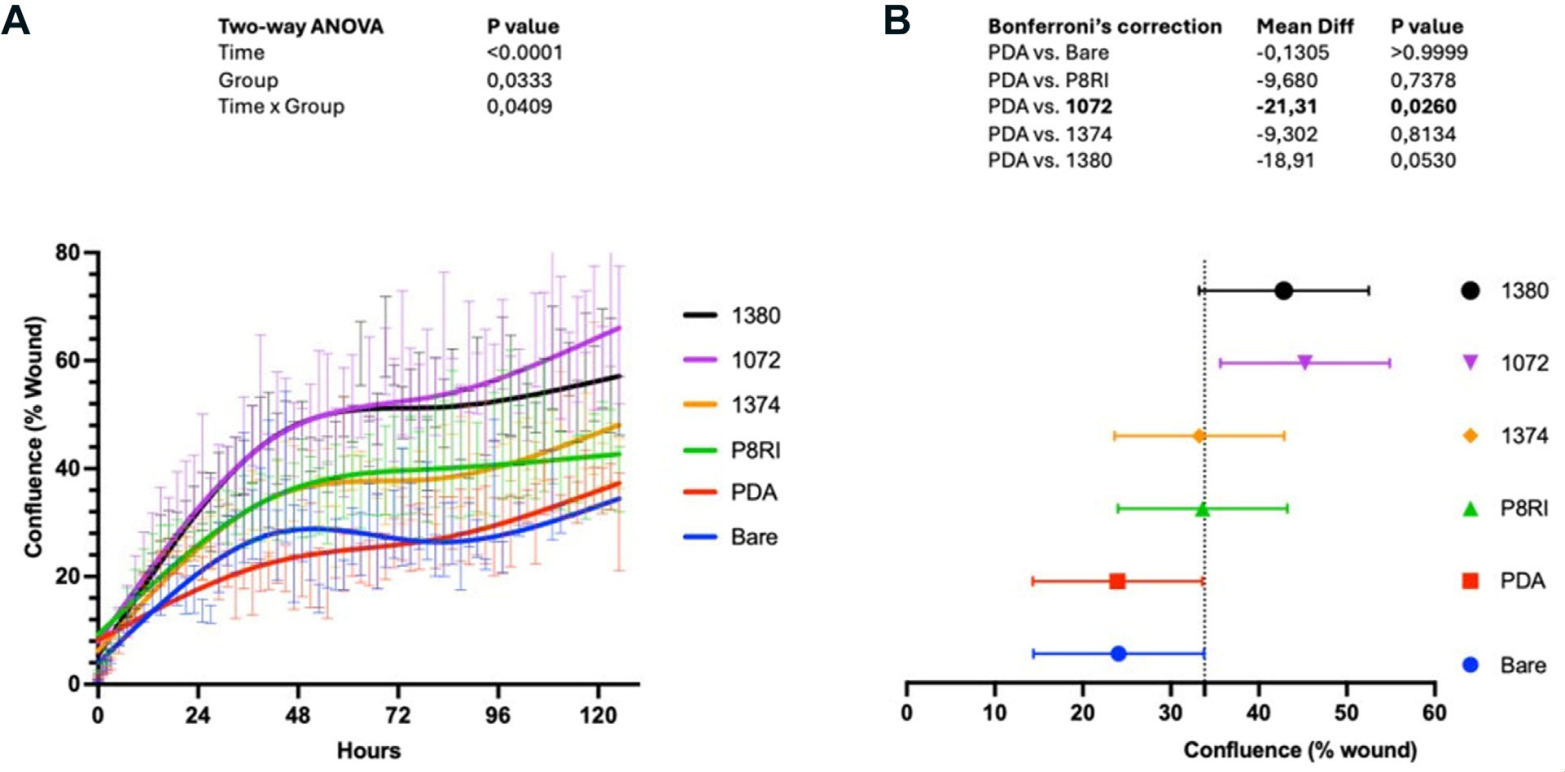
Assessment of wound coverage (scratch test). **(A)** Analysis of the migration and growth of HAEC within the wound over 6 days, observed through sequential scans taken every 3 hours. Differences in wound coverage, assessed using a two-way analysis of variance with Bonferroni's correction for multiple comparisons, are presented. Group effect is expressed as the confluence percentage of the wound, and Time effect is expressed in hours for each indicated group. Lines represent the mean, and error bars indicate the standard error of the mean (n = 3 per group). **(B)** A nested one-way analysis of variance of the mean dataset reveals that among all the tested CD31 peptides, both SP1380 (with an 18.91% increase) and SP1072 (with a 21.31% increase) exhibit the most effective specific effects compared with polydopamine (*PDA*). The difference between SP1072 and PDA is the only statistically significant one (*P* < .05).

Because all the trans-homophilic peptides consistently promoted a physiological EC phenotype, we chose to advance to the functional testing phase using monomers derived from the first three groups. This selection was driven by the relative simplicity of monomer synthesis in contrast with the more complex dimer synthesis in groups IVa and IVb. This decision was made with scalability in mind for future production. We immobilized SP1374 (group I), SP1072 (group II), and SP1380 (group III) peptides onto 96-well plates for use in the scratch test using the previously described three-step dip-coating method. The analysis was performed using Incucyte Cell Migration Scratch Wound Analysis software. As depicted in Fig 2, *A*, all the peptides demonstrated consistent and enhanced wound coverage over time in vitro. However, only the effect of SP1072 peptide in terms of confluence (wound coverage) reached statistical significance compared with bare and PDA-coated surfaces in nested one-way analysis of variance analysis (Fig 2, *B*). Consequently, we selected the SP1072 peptide for coating clinical-grade CoCr stents for in vivo testing.

### In vivo effects of the camouflaging coating on stents implanted at abdominal aortic sites

**Fig 3:**
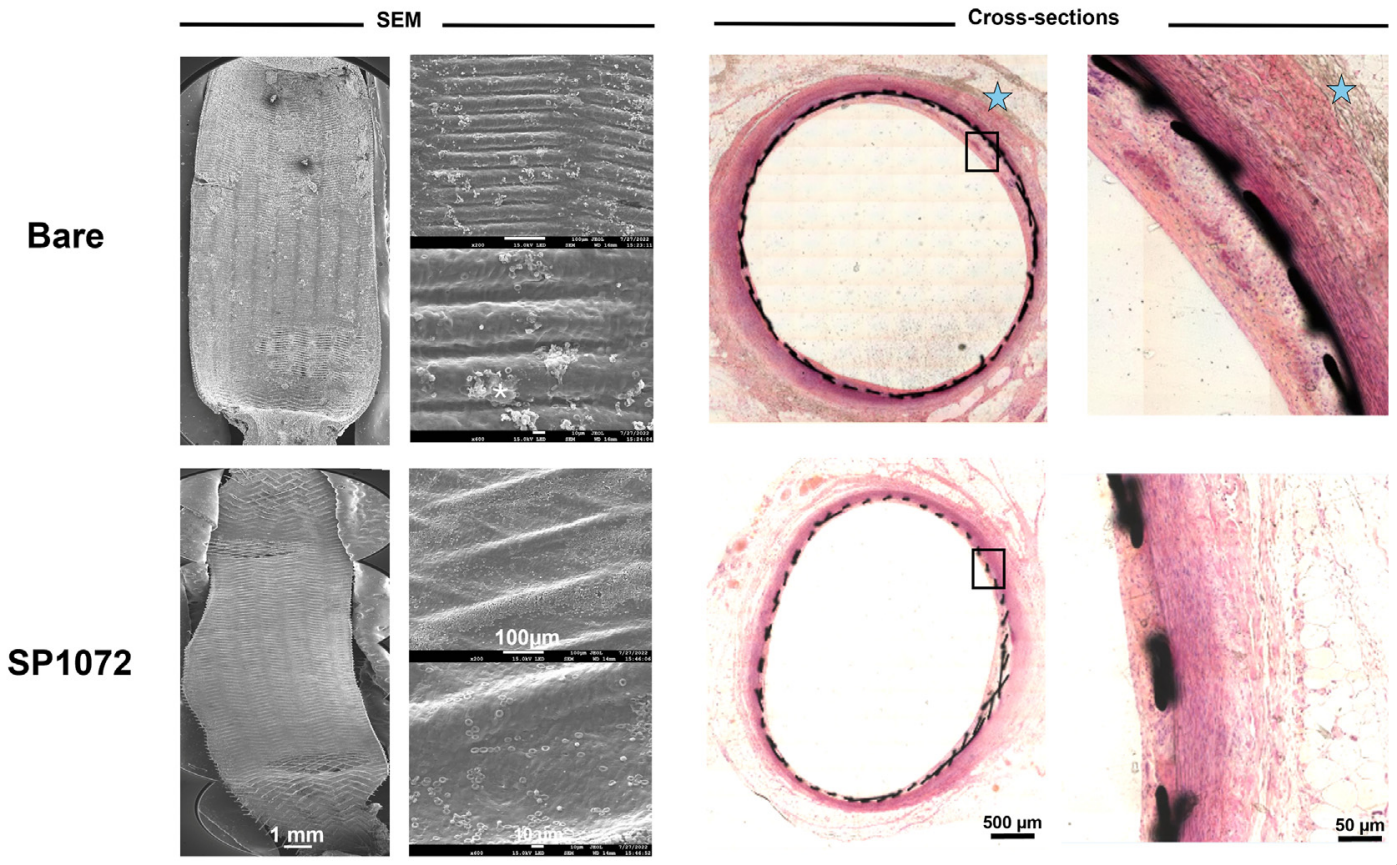
In vivo results 7 days after implantation of bare and CD31 D1-coated (SP1072) nitinol stents. Scanning electron microscopy (*SEM*) imaging show fair endothelial coverage on both types of stent surface, but some areas demonstrated varying degrees of erythrocytes, inflammatory cells, and platelets adhering to the stent surface and these findings were consistently more frequent on control Bare stents. Cross-sections of stented innominate artery showed discrete neointima formation and adventitial inflammation only in the bare group (blue stars). The scale bars at the bottom apply to all panels in the corresponding column.

**Fig 4:**
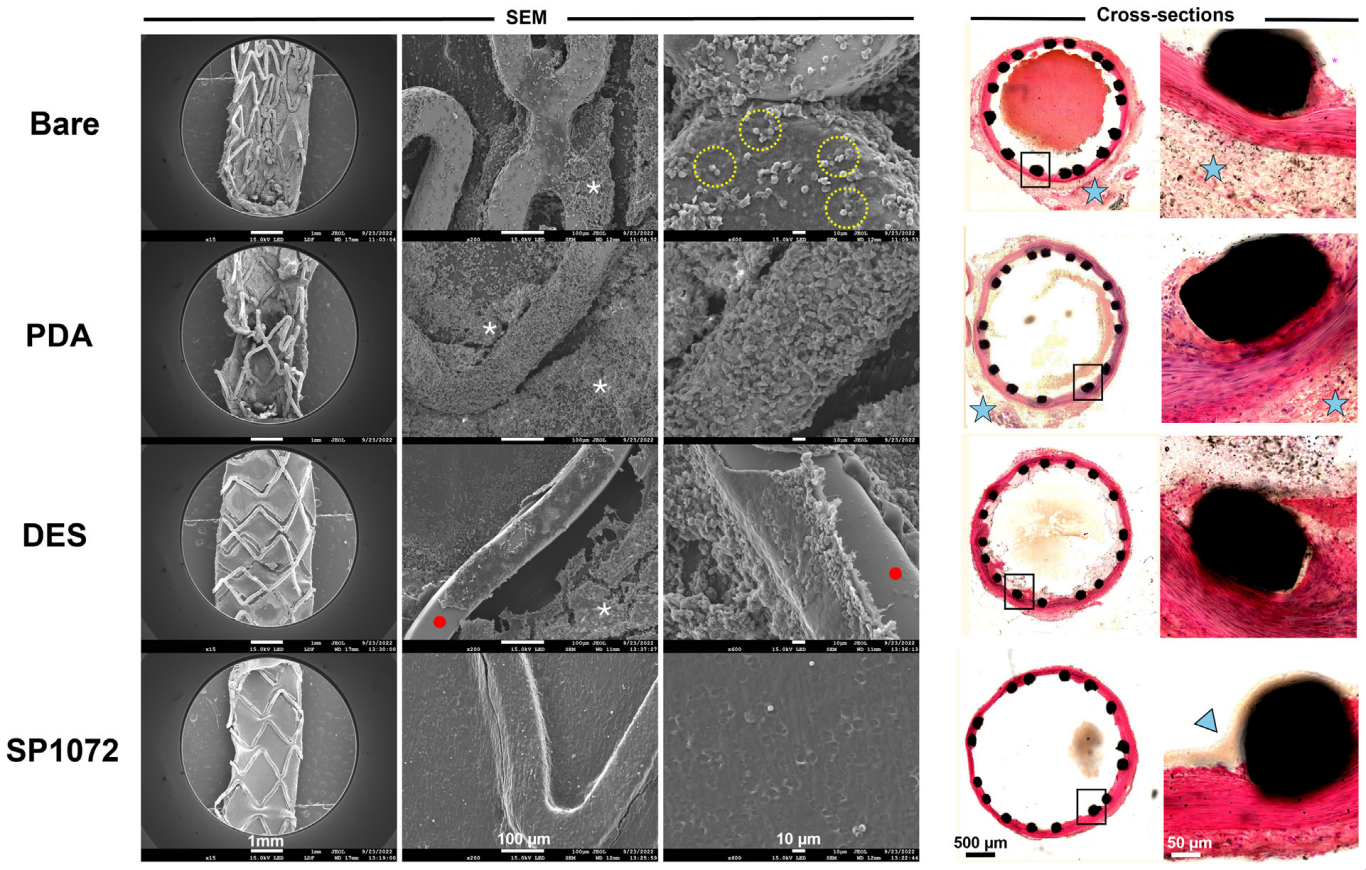
CD31 D1-coated CoCr stents outperform polydopamine (*PDA*), drug-eluting stents (*DESs*), and bare metal stents (BMSs) by day 7 in terms of endothelial coverage and thromboinflammation. Scanning electron microscopy (*SEM*) images show a neoendothelium associated with platelet/fibrin aggregates (white asterisks) and several blood cells (mainly leukocytes) over the stented luminal area of bare, PDA, and DESs. The leukocytes are tightly adhering (pseudopods over the surface) and many of them are infiltrated in the subendothelial space (beneath the endothelial layer, yellow dashed circles). PDA stents were covered with an uneven endothelium showing intense platelet/fibrin aggregates (white asterisks). DESs show several areas not covered with endothelium (red dots). The latter seems to be covered with several blood cells (mainly platelets and erythrocytes). CD31 D1-coated stents (SP1072) are mostly covered with a smooth and clean endothelium. A few resting, round-shaped leukocytes and erythrocytes appear on the surface, supposedly adhering post mortem. Resin cross-sections stained with hematoxylin and eosin show dense adventitial inflammation (blue stars) and platelet/fibrin aggregates next to the struts (black structure) of bare and PDA stents, whereas both adventitial inflammation and platelet/fibrin aggregates were moderate on DESs. SP1072 stents showed no adventitial inflammation and an even and complete endothelialization of the luminal side of the stent struts (“jelly”, thin grey layer, blue arrowhead) without fibrin, platelets, or leukocytes on the luminal side. The scale bars at the bottom apply to all panels in the corresponding column.

**Fig 5:**
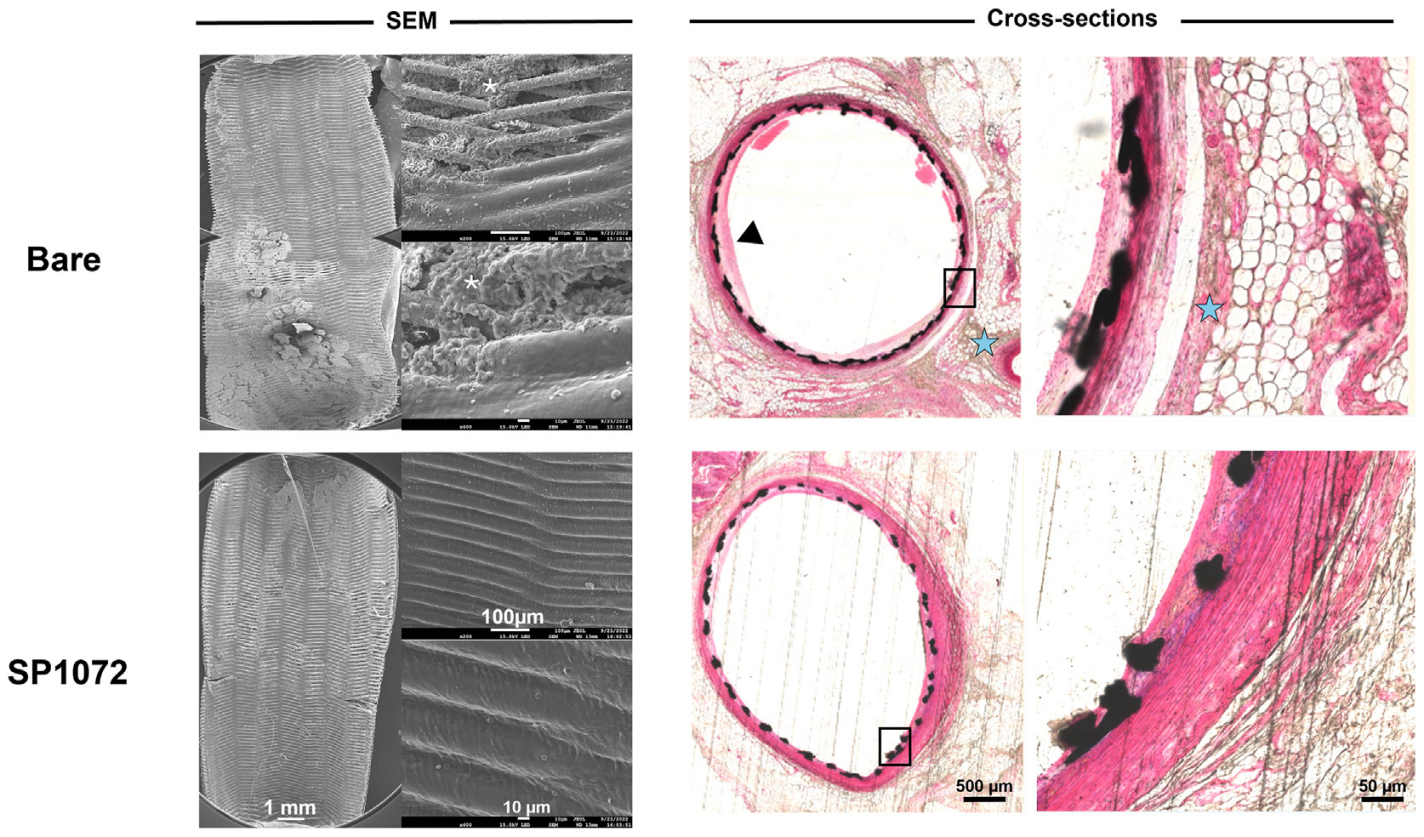
In vivo results 30 days after intra-aortic implantation of bare and CD31 D1-coated (SP1072) nitinol stents. Scanning electron microscopy (*SEM*) images of the luminal face of the stented aorta showed numerous clots in topical areas associated with platelet/inflammatory cells aggregation in the bare stents (white asterisk) whereas a smooth endothelial coverage with only minimal amounts of adhering erythrocytes was observed in the SP1072 stents. Cross-sections of stented innominate artery showed discrete neointima formation (black arrowhead) and adventitial inflammation (blue stars) only in the bare group. The scale bars at the bottom apply to all panels in the corresponding column.

**Fig 6:**
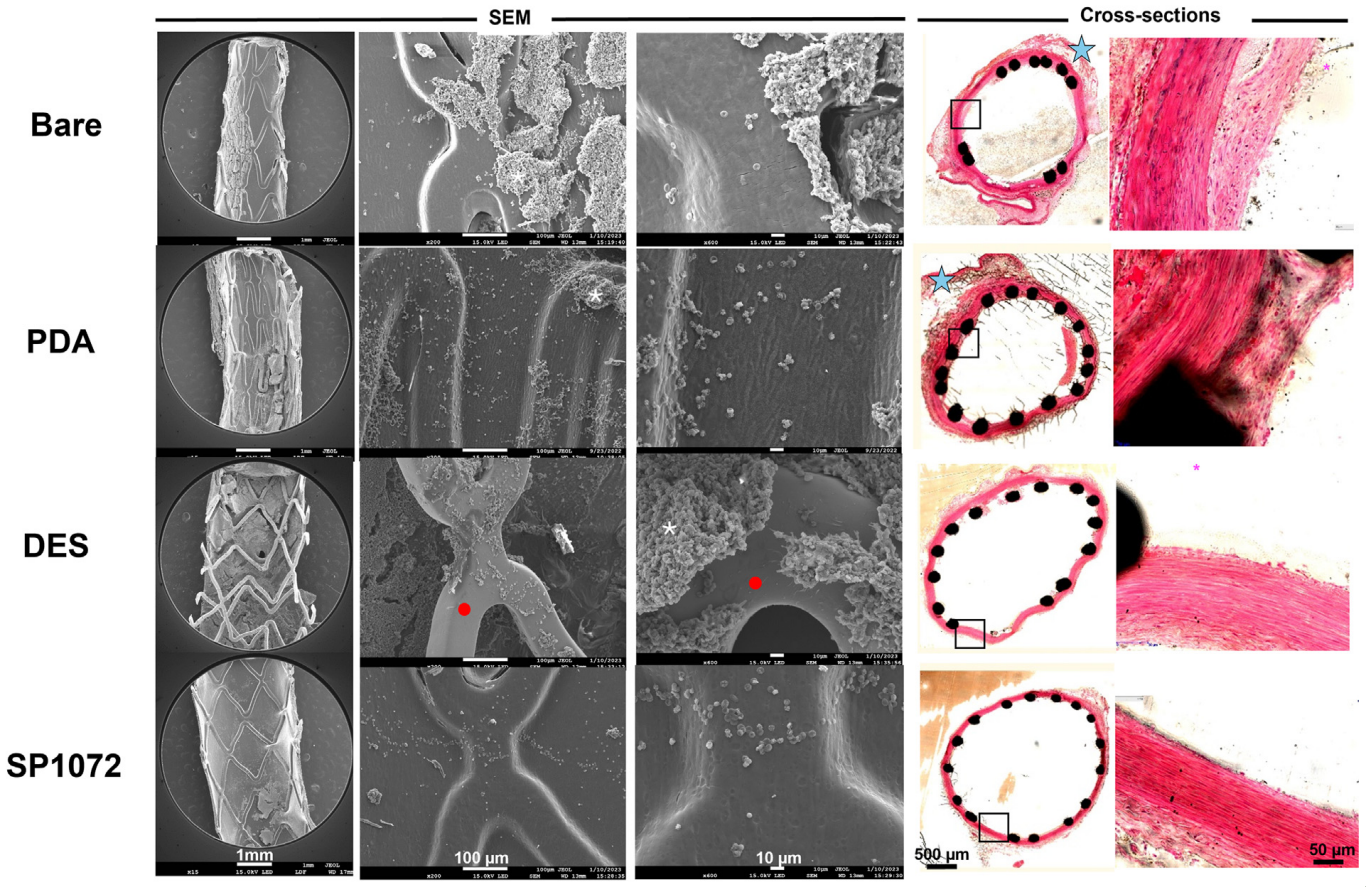
Persistent effect of CD31 D1-Coated (SP1072) CoCr stents by day 14. Scanning electron microscopy (*SEM*) images of the luminal face of the bare, polydopamine (*PDA*), and drug-eluting (*DES*) stents showed complete endothelialization with various extents of fibrin/platelet aggregates (white asterisks) and discrete lack of endothelial coverage on DESs (red dots); SP1072 stents were covered by a smooth endothelium without fibrin/platelet aggregates. Cross-sections showed mild neointima formation on the stented luminal area of the bare and PDA stents. DES and SP1072 stents showed a smooth intimal surface devoid of fibrin/platelet aggregates and neointima formation. The scale bars at the bottom apply to all panels in the corresponding column.

**Fig 7:**
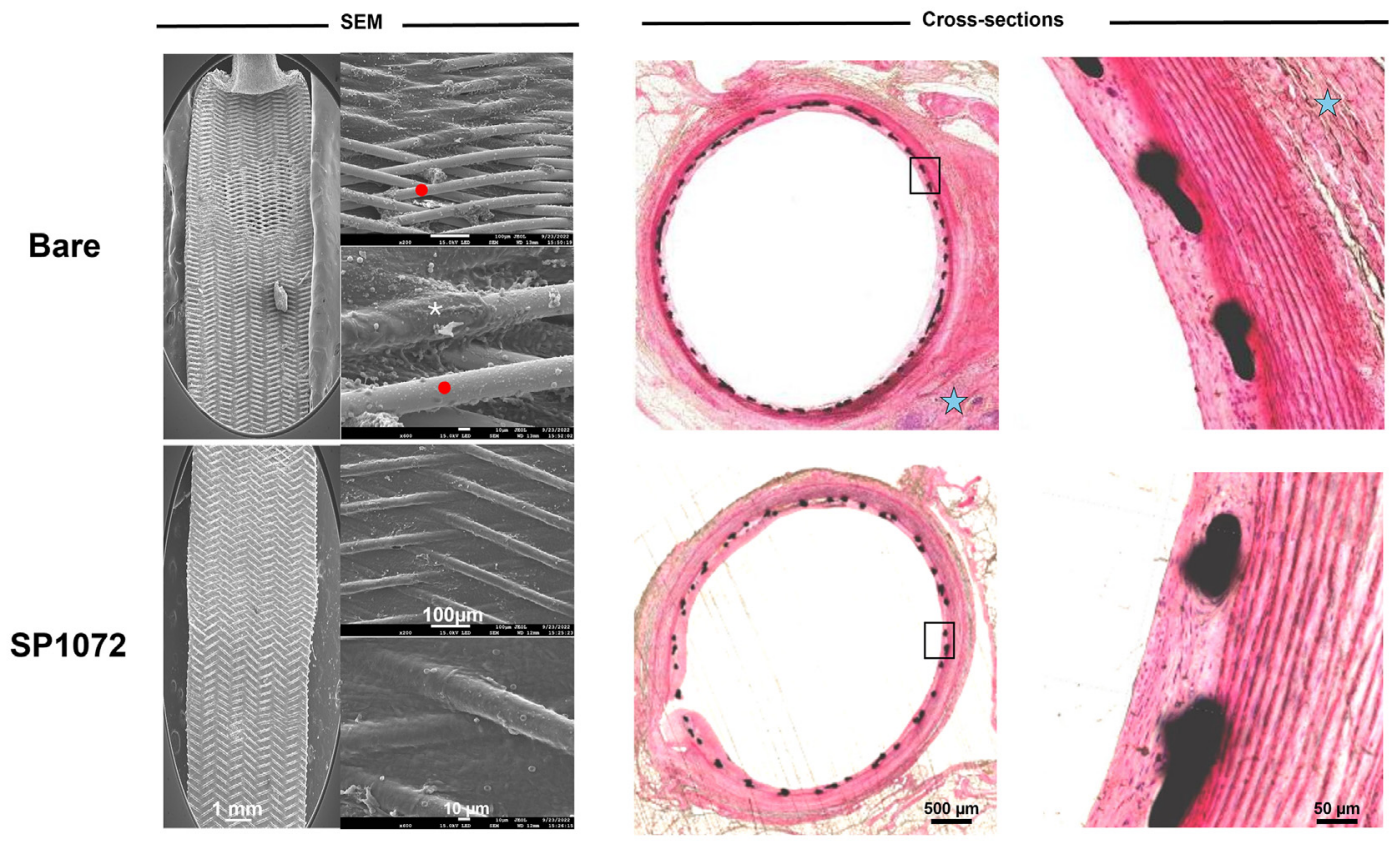
In vivo results 60 days after intra-aortic implantation of bare and CD31 D1-coated (SP1072) nitinol stents. Scanning electron microscopy (*SEM*) images of the luminal face of the implanted showed the presence of platelet-leukocyte aggregates (white asterisks) and incomplete endothelialization (red dots) in topical areas of the bare stents. The endothelial coverage was uniform and smooth, devoid of inflammatory cells and platelets aggregates without adventitial inflammation in the SP1072 stents. Cross-sections of stented innominate artery showed discrete adventitial inflammation (blue stars) only in the bare group. The scale bars at the bottom apply to all panels in the corresponding column.

**Fig 8:**
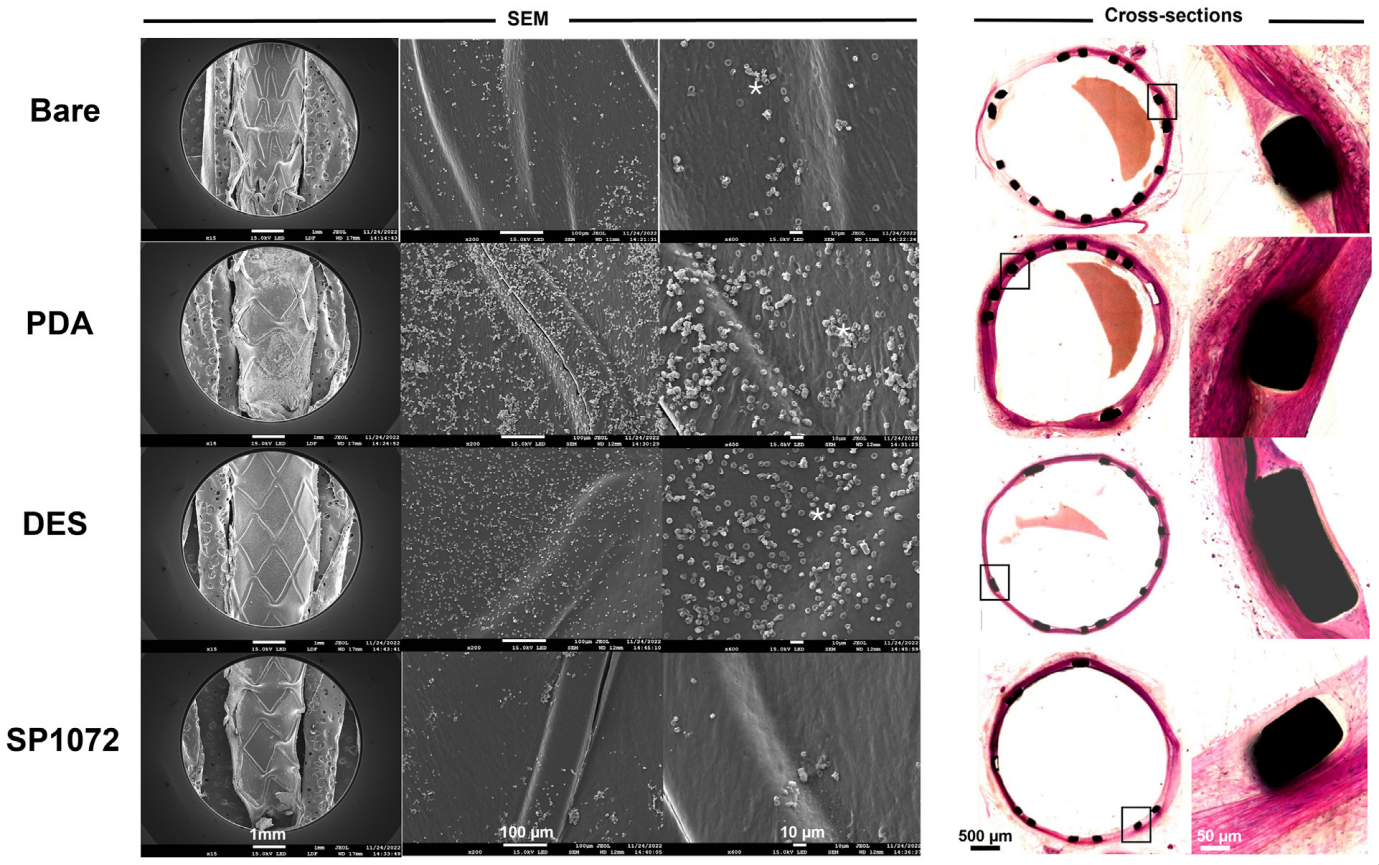
CD31 D1-coated CoCr stents outperform polydopamine (*PDA*), drug-eluting (*DES*), and bare metal stents (BMSs) by day 30 in terms of endothelial coverage and thromboinflammation endothelialization was complete in all stents. Discrete fibrin/platelet deposits (white asterisks) were observed on bare, PDA, and DES stented arteries, whereas they were virtually absent on CD31 stent-covering endothelium. Mild neointima formation was observed on the cross-sections of bare metal and PDA stents, whereas neointima formation was virtually absent from both DES and SP1072 stents.

**Supplementary Fig 3 (online only):**
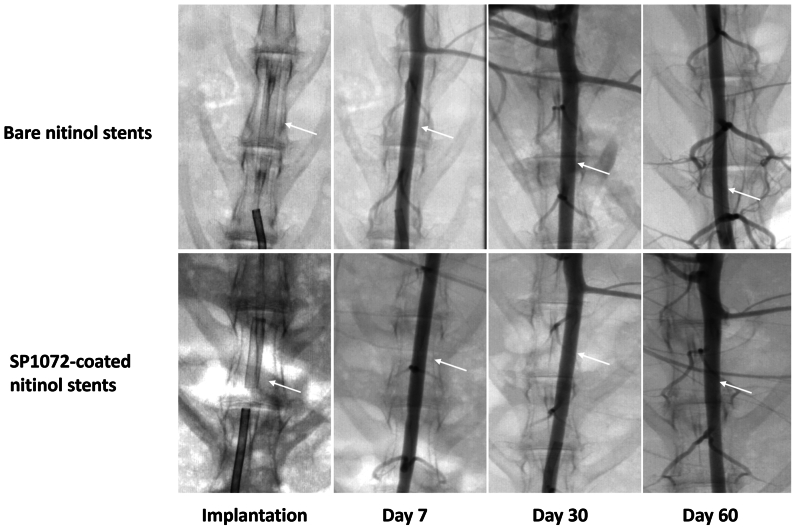
Angiographic analysis of nitinol devices. The locations of stents in the infrarenal aorta of rabbits on the implantation day and their respective position in pre-explantation angiograms at different timepoints are shown by the arrows. The angiograms showed correct expansion and patency of all devices at each different timepoint.

**Supplementary Fig 4 (online only):**
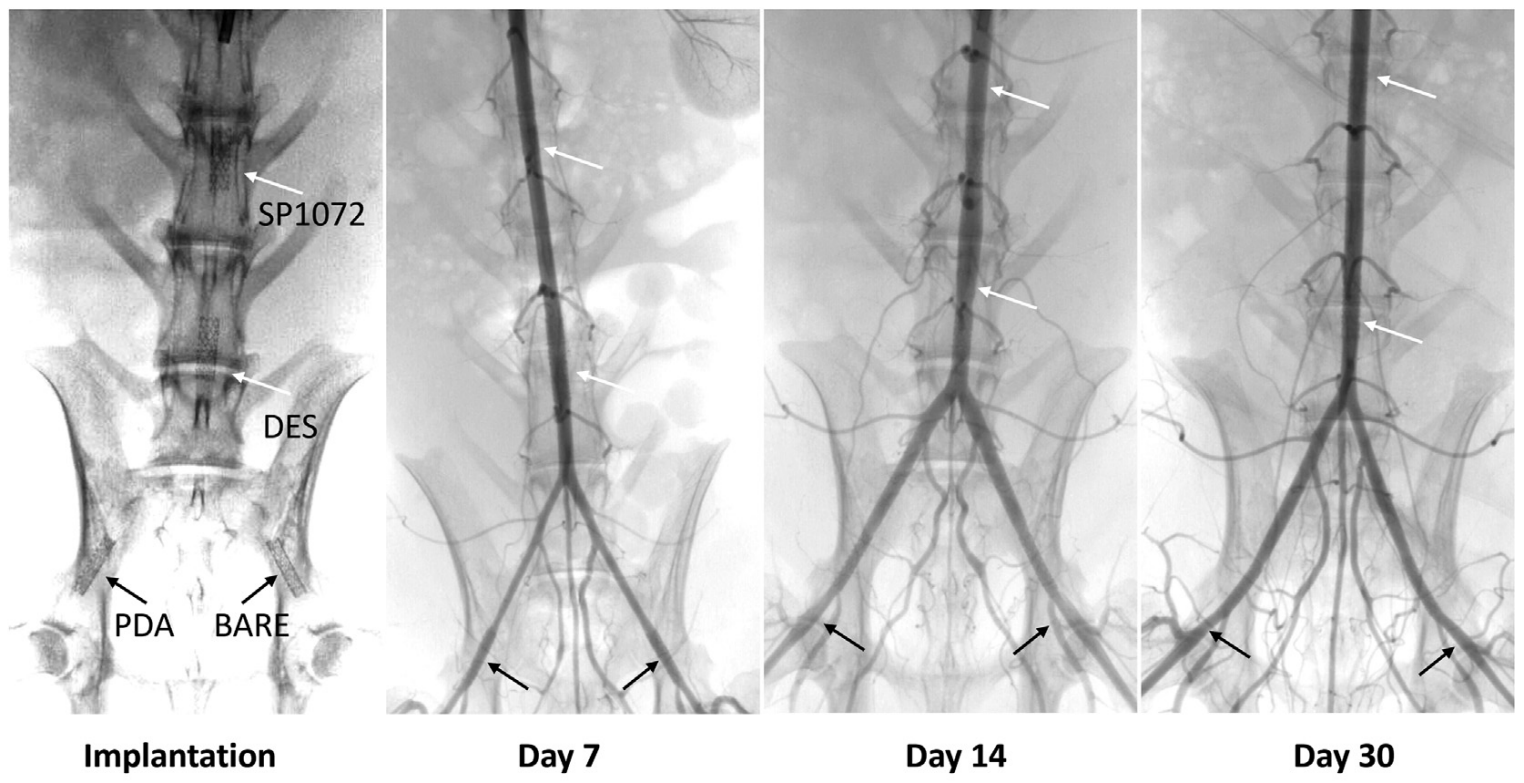
Angiographic analysis of cobalt-chromium (CoCr) devices. The respective location of each of the four types of stents on the implantation day and their respective position in pre-explantation angiograms at different timepoints are shown by the arrows. SP1072 and drug-eluting stents (DESs) were implanted in the proximal and distal infrarenal aorta, whereas polydopamine (PDA) and bare metal stents (BMSs) were implanted in the right and left iliac arteries of rabbits, respectively. The angiograms showed correct expansion and patency of all devices at different timepoints.

All stents were implanted successfully and remained patent until explantation, as confirmed by repeated angiography in both study arms (Supplementary Figs 3 and 4, online only). Scanning electron microscopy analysis consistently demonstrated that CD31-coated nitinol and CoCr stents were fully covered by a smooth endothelium with minimal blood element adhesion (see Fig 3, Fig 4, Fig 5, Fig 6, Fig 7, Fig 8). In contrast, control devices exhibited variable platelet and leukocyte adhesion, and endothelial coverage was delayed with DES, consistent with most recent human reports.^16^ Resin cross-section analysis revealed a healthy endothelium on CD31-coated stents, whereas BMSs often displayed signs of local thromboinflammation around the endothelialized struts. Notably, CD31-coated stents consistently showed no signs of medial damage or adventitial inflammatory reaction at 7, 14, 30, and 60 days after implantation. Conversely, despite full endothelial coverage and administration of DAPT, platelet aggregation and neointima formation were evident in resin cross-sections of BMS struts (see Fig 3, Fig 4, Fig 5, Fig 6, Fig 7, Fig 8). However, no significant lumen loss was observed on angiograms owing to the study's design, which did not aim to evaluate stenosis effects in the absence of mechanical damage at the time of implantation in healthy rabbit arteries.

## Discussion

In this study, we describe the different steps leading to the development of a novel biomimetic coating, starting from the design of peptides inspired by human CD31 trans-homophilic interactions to the functionalization of full-scale, clinical-grade nitinol and CoCr stents. The CD31-derived coating developed in the present study enhanced the physiology of ECs growing onto coated surfaces in vitro significantly compared with control groups. Among the peptides tested, SP1072 (group II, sequence: YKSTVIVNNKEKTTAE-PEG4-K(N3)-CONH2), exhibited the most favorable in vitro properties and was consequently selected to coat clinical-grade 3 × 40-mm self-expanding nitinol and 2.5 × 20.0-mm balloon-expandable CoCr stents. In vivo experiments demonstrated promising early and mid-term results, with CD31-coated devices seamlessly integrating into the arterial wall without thromboinflammatory signs, in contrast with BMS and DES stents, ≤30 days after implantation.

This strategy is in line with recent research in the field of endovascular devices, shifting its focus from targeting vascular smooth muscle cells with active stent drugs^17^ toward promoting EC growth to rapidly achieve neoendothelialization and comprehensive coverage of the device.^18^ Indeed, despite the significant relative risk reduction for restenosis and target lesion revascularization offered by DESs compared with BMSs in the context of peripheral artery disease,^4^^,^^19^ various unresolved issues persist. These issues include a limited understanding of the arterial response to injury after DES implantation, the course of late stent thrombosis, and determining the optimal duration of DAPT. Although DAPT has demonstrated efficacy in decreasing DES thrombosis risk and prolonged survival after lower extremity revascularization,^20^ it is closely tied to life-threatening bleeding complications.^21^

Several alternative approaches to DES emerged, including the design of coatings with geometries that favored endothelialization,^22^ the capture of ECs, and, more recently, the controlled release of EC growth factors such as nitric oxide^23^ or vascular endothelial growth factor.^24^ Notably, the concept of capturing ECs using antibodies targeting endothelial precursor cells like CD34,^25^ vascular endothelial cadherin,^26^ or CD133^27^ gained interest in stent coatings. Although such proendothelialization strategies exhibit promising results in preclinical studies, the incapacity of the captured ECs to acquire a physiological phenotype likely accounts for the lack of benefits and even an increased risk of adverse outcome compared with DESs, with no significant difference observed when compared with BMSs.^28^

In essence, achieving endothelialization alone is not sufficient for success. Indeed, even BMSs can achieve complete endothelialization within 1 week,^29^ yet they exhibit a stent thrombosis rate comparable with DESs, where efficient endothelialization may be lacking, as documented by clinical studies comparing BMSs and DESs in femoropopliteal artery stenting.^30^ This finding underscores the importance of the functional role of ECs, which go beyond being a mere barrier. Under physiological conditions, they play critical roles in maintaining vascular homeostasis, including regulating vascular tone, inhibiting platelet adhesion, modulating leukocyte activation, promoting relaxation, and inhibiting vascular smooth muscle cell proliferation.^31^

When subjected to pathological stressors, such as the presence of foreign bodies like stents, ECs activate and release tissue factor, initiating an extrinsic coagulation cascade and promoting thrombin generation and platelet aggregation.^32^ This result highlights the pressing need for innovative approaches that can replicate a physiological endothelium on stent surfaces, rather than merely promoting the presence of any endothelium. CD31 molecules expressed on healthy cell surfaces play a pivotal role in this process, engaging in trans-homophilic interactions involving D1 and D2.^13^ These interactions trigger a cascade of events, including the cis-homophilic interaction between CD31 molecules on each cell^33^ that is crucial for driving CD31's intracellular signaling functions, eventually leading to the detachment of individual cells within the blood suspension and the transmission of vital physiological signals at the EC-cell junctions.^8^ Interestingly, although the amino acid sequence of SP1702 (group II) partially overlaps with that of SP1379 (group IVa), the benefits observed with the latter are less convincing. This apparent discrepancy may be attributed to their specific 3D conformations. Specifically, SP1072 (group II) peptide reproduces one arm of the beta sheet formed by D1, whereas SP1379 (group IVa) is a heterodimer formed by a sequence equivalent to SP1072 combined with an equivalent of SP1374 (group I), providing two additional sides to the beta sheets forming D1.

Our aim was to explore various potential surfaces that could offer a trans-homophilic platform, based on the crystal structure demonstrating that the two D1 and D2 of the trans-homophilic interacting CD31 molecules are packed in a face-to-face antiparallel pattern with the side face of one β sheet. Groups I and II reproduce the two sides of the beta sheet formed by D1, potentially offering interacting surfaces to the beta sheet formed by D2 of adjacent CD31 molecules. Group III reproduces the exposed side of the beta sheet of D2, serving the same purpose. Additionally, the whole D1 might be required for interaction with D2 of adjacent CD31 molecules, a scenario tested with peptides of group IVa. Finally, group IVb reproduces the interaction between the facing sides of the beta sheets of D1 on one cell and of D2 on the other cell, supporting engagement between the remaining free D2 on the first cell and D1 on the other cell.

Our in vitro experiments suggest that the different 3D conformations influence the mimetic function of the coated surface, with the lateral side of the beta sheet formed by D1 (group II) demonstrating the best efficacy.

In the realm of endovascular devices for treating peripheral arterial disease, the application of CD31 trans-homophilic peptides to mimic a healthy endothelium holds immense promise. This innovative approach could not only could spare the use of DAPT, but also render the use of anti-proliferative drugs unnecessary, because the growth factors responsible for neointima proliferation are released by chronically activated leukocytes and platelets at the site of implantation, and the activation of platelets and leukocytes at the contact with trans-homophilic CD31-coated surfaces is abolished inherently. The use of CD31 trans-homophilic peptides as a coating, therefore, introduces a unique drug-free, multifaceted approach that addresses all aspects of potential endovascular device post-implantation complications effectively.

### Limitations

In acknowledging the promising results presented in this study, it is essential to recognize several limitations. First, although our in vivo experiments provided valuable insights into the potential of the endothelial-mimetic device surface, they relied primarily on qualitative analyses. Second, although our primary aim was to investigate stent healing, it is crucial to extend this analysis to assess its impact on subsequent outcomes, such as myointimal hyperplasia. Longer studies with sample sizes sufficient to draw quantitative data for a direct comparison between experimental groups are necessary to complement these qualitative findings and directly evaluate the efficacy and reproducibility of the coating's performance on both arterial healing and myointimal hyperplasia.

Additionally, the selection of the peptide for the in vivo experiments was based on functional screening conducted in vitro. These experiments indicated that different 3D conformations influence the mimetic function of the coated surface, with the lateral side of the beta sheet formed by D1 (group II) demonstrating the best efficacy. However, it is important to note that these experiments were conducted under static conditions, and the in vivo testing was limited to the most promising option to minimize animal use in this pioneering study. Therefore, further exploration is warranted to investigate whether other conformations of the mimetic peptides, apart from group II, may have beneficial effects when evaluated under flow conditions, incorporating blood hemodynamic forces and circulating elements such as leukocytes and platelets.

Moreover, although the coating remained intact for ≤1 year under static conditions in our experience (data not shown), assessing its long-term durability in physiological flow conditions is essential. Hence, future research efforts should focus on incorporating quantitative assessments to gain a comprehensive understanding of the functional outcomes associated with different peptide coatings. Long-term durability assessments under physiological flow conditions are crucial to ensure the translational potential of these findings.

Finally, although our rabbit results are promising, it is important to acknowledge that the response to stent implantation in humans can be influenced by various factors, including the complexity of the underlying disease, the patient's overall health status, and the presence of comorbid conditions. Therefore, interpreting the observed stent behavior in the aorta and iliac arteries of healthy rabbits in the context of translation to patients with aortoiliac occlusive disease and infrainguinal disease requires careful consideration. Although animal studies provide valuable insights, clinical research is necessary to validate the applicability of these findings in real-world patient populations. Addressing these limitations will pave the way for further advancements in understanding the therapeutic potential of CD31-mimetic peptide coatings in vascular stent applications.

## Conclusions

Our findings suggest that using peptides replicating the membrane-distal portion of CD31, which is prominently exposed on the inner side of healthy vessels, can simulate the surface of a healthy endothelium. This approach effectively prevents the deposition and activation of blood platelets and leukocytes, which are abnormally prolonged by the presence of a foreign body at sites of endovascular stent implantation, and promotes the acquisition of physiological phenotype by the ECs covering the surface.

The endothelial-mimetic coating of CD31 D1 used in this study can be considered as a self-healthy surface, offering the potential to achieve rapid arterial healing. The versatile mussel-inspired and click-chemistry dip-coating method can be applied to various blood-contacting devices, including stents, occluders, and valve bioprostheses. Importantly, this strategy may eliminate the need for drugs to manage platelet activation or foreign-body reactions.

Further studies are necessary to investigate the efficacy and safety of CD31 D1 coatings on a larger scale, as well as their long-term durability and potential clinical applications. This study provides a promising step toward the development of more effective and biocompatible endovascular devices.

## Author Contributions

Conception and design: JS, CS, AN, CB, GC

Analysis and interpretation: JS, CS, BH, RS, IL, EB, YC, AN, CB, GC

Data collection: JS, CS, BH, IL, EB, GC

Writing the article: JS, CS, CB, GC

Critical revision of the article: JS, CS, BH, RS, IL, EB, YC, AN, CB, GC

Final approval of the article: JS, CS, BH, RS, IL, EB, YC, AN, CB, GC

Statistical analysis: JS, CS, BH, EB, YC, GC

Obtained funding: AN, CB, GC

Overall responsibility: GC

JS and CS contributed equally to this article and share co-first authorship.

## Funding

This research was funded by the “pré-maturation” program of the University Paris Cité (StratEx, project “BEND” 2020-2022), by the RHU iVASC (ANR-16-RHUS-00,010) and by a research collaborative contract between Inserm-Transfert and Alchimedics S.A.S. C.S.’s salary is supported by the French National Research Agency (ANR) as part of the future investment program in tegrated into France 2030, under grant agreement No. ANR-18-RHUS-0001.

## Disclosures

C.S., I.L., C.B., A.N., and G.C. are the inventors of two patent applications related to the work presented in this article. G.C., A.N., C.S., E.B., and R.I. (PCT/EP2022/084,241) Biomimetic Coating for Endovascular Stent (INSERM, University Paris Cité, University Sorbonne Paris Nord, INSERM). G.C. A.N., C.S., C.B., and I.L. I. (PCT/IB2022/000,684) Biomimetic Coating for Endovascular Stent (AlchiMedics S.A.S., INSERM, University Paris Cité, University Sorbonne Paris Nord).

## Appendix (online only)

### Supplementary Methods

#### Peptide design

Group I peptides were designed to mimic the region of human CD31 D1 spanning His71-Ser87, or Gln70-Lys89. The consensus sequence QHXXLFYKDDXXFYNISSXX was derived, with certain amino acids (aa) allowing for variation. Cysteines were incorporated to form a disulfide bond and a cyclic structure. The following peptides were included in group I: SP722, SP745, SP765, SP1374, and SP1375.

Group II peptides were designed to imitate the region of human CD31 D1 corresponding with Tyr107-Glu122 or 106-124 aa. Cysteines were incorporated to form a disulfide bond and a cyclic structure. The consensus sequence YKSTVIXNNKEKTTXE was established with specific aa variations in the individual peptides. Group II peptides included SP1072 and SP1376.

Group III peptides were created to mimic the region of human CD31 D2 from Pro133-Lys158. Val135hCys and Cys152hCys were introduced to form disulfide bonds and cyclic structures. The consensus sequence, PXCTLDKKEXIQGGXVXVNCSVPEEK, was developed, with particular aa allowing for variability. The peptides in group III included SP1071 and SP1380.

Group IVa peptides consist of heterodimers, combining peptides from group II and group I through covalent linkage. Group IVb peptides were also heterodimers, combining peptides from group III and group I. Peptides SP1379 and SP1383 were part of groups IVa and IVb, respectively.

Such peptide design, sequences and applications were patented under the following registration numbers: PCT/EP2022/084,241 and PCT/IB2022/000,684.

#### Peptides synthesis and structural analysis

All peptides were synthesized using standard solid phase peptide synthesis protocols with Fmoc/t-Bu chemistry and dimethylformamide as the solvent. Owing to their high sequence similarity, the individually designed peptides were screened in vitro to identify potential candidates for coating stents in future in vivo applications, based on their proven ability to induce a prophysiological phenotype in ECs.

To assess their structural similarity to the endogenous protein from which the sequences were derived, the peptides were dissolved in a phosphate buffer (pH 6.8) at a concentration of 5 mmol/L. Raman spectra were acquired by exciting approximately 50 μL of the sample solutions in Suprasil quartz cells (5-mm path length) using the 488-nm line emitted by an Ar + laser (Spectra-Physics Inc, Andover, MA). Scattered light was analyzed using a Jobin-Yvon T64000 spectrograph, equipped with a 1200 grooves/mm holographic grating and a holographic notch filter, operating in a single macro configuration. The effective spectral slit width was set at approximately 5 cm^−1^.

#### Stent implantation

Stent implantation was performed under general anesthesia (acepromazine 0.5 mg/kg, isoflurane 1.5%). Intramuscular administration of ceftriaxone was performed (0.1 g/kg) along with heparin sodium (150 IU/kg) immediately before implantation. Bilateral femoral cutdown were performed and stents were all implanted under fluoroscopy guidance. All stents were preoperatively sterilized using beta radiations (25 kGy). After postimplantation angiographic control, the femoral access was closed in a standard fashion. Animals received oral acetylsalicylic acid 5 mg/kg and clopidogrel 5 mg/kg once a day, from 3 days before surgery to the postoperative end point. Arterial angiograms were repeated at the end of the study period in each animal, immediately before explantation, to assess main and collateral vessel patency of stented aortas and iliac arteries.

#### Histopathology analysis

We examined the entire luminal surface of one implanted arterial segment at each time point for each group to assess endothelial stent coverage and the presence of platelet/leukocyte deposits on scanning electron microscopy en face preparations. Despite using different sizes of devices for aortas and innominate and iliac arteries, this method facilitated a consistent evaluation of endothelialization across the entire luminal surface of all the stents, regardless of their dimensions. Resin cross-sections from nitinol samples implanted in the innominate artery or in the aortas and iliac arteries of matched sets of experiments within the CoCr study arm were utilized to evaluate the presence of neointima and the morphology of the outer arterial layers, including peristrut fibrin deposits and/or leukocyte infiltration, media integrity, and adventitial inflammation, using hematoxylin staining.

## Appendix

Additional material for this article may be found online at www.jvsvs.org.
